# Ruptured Urinary Bladder Diagnosed by Point-of-care Ultrasound

**DOI:** 10.5811/cpcem.2021.10.54410

**Published:** 2021-12-15

**Authors:** John C. Bates, Victoria Kneller, Layla Abubshait

**Affiliations:** Einstein Healthcare Network, Department of Emergency Medicine, Philadelphia, Pennsylvania

**Keywords:** POCUS, ultrasound, bladder rupture

## Abstract

**Case Presentation:**

We describe a case of abdominal pain in a male patient who performed daily self-catheterization and developed a ruptured urinary bladder, which was diagnosed at bedside in the emergency department with point-of-care ultrasound (POCUS).

**Discussion:**

Ruptured urinary bladder is commonly associated with blunt abdominal trauma. It is a rare complication of Foley catheter insertion. These images demonstrate that POCUS can be used as a screening tool to evaluate for bladder rupture when clinically suspected.

## CASE PRESENTATION

An 83-year-old male with a history of metastatic prostate cancer requiring daily self-catheterization presented to the emergency department (ED) complaining of abdominal pain. His abdominal pain was mild and intermittent over the prior several days, without any aggravating or alleviating factors. The patient had been self-catheterizing five times per day, without resistance or gross hematuria. On arrival to the ED his vital signs were stable. Physical exam showed an elderly gentleman in pain with pale conjunctiva. His abdomen was soft, non-distended, and tender over the suprapubic area.

**Image 1:**
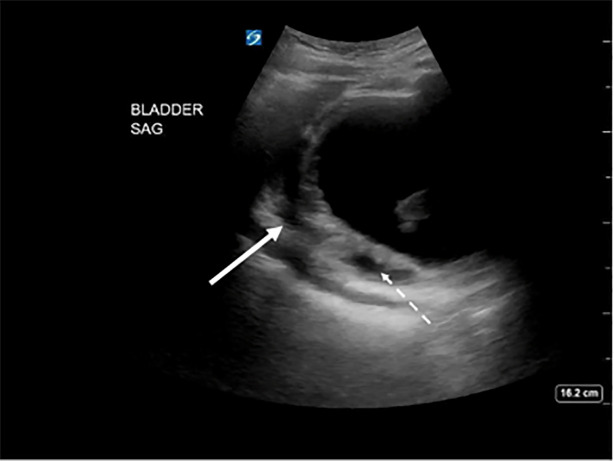
Sagittal view of the bladder on point-of-care ultrasound showing posterior, fluid-filled loculations (solid arrow) and wall thickening (dashed arrow).

**Image 2:**
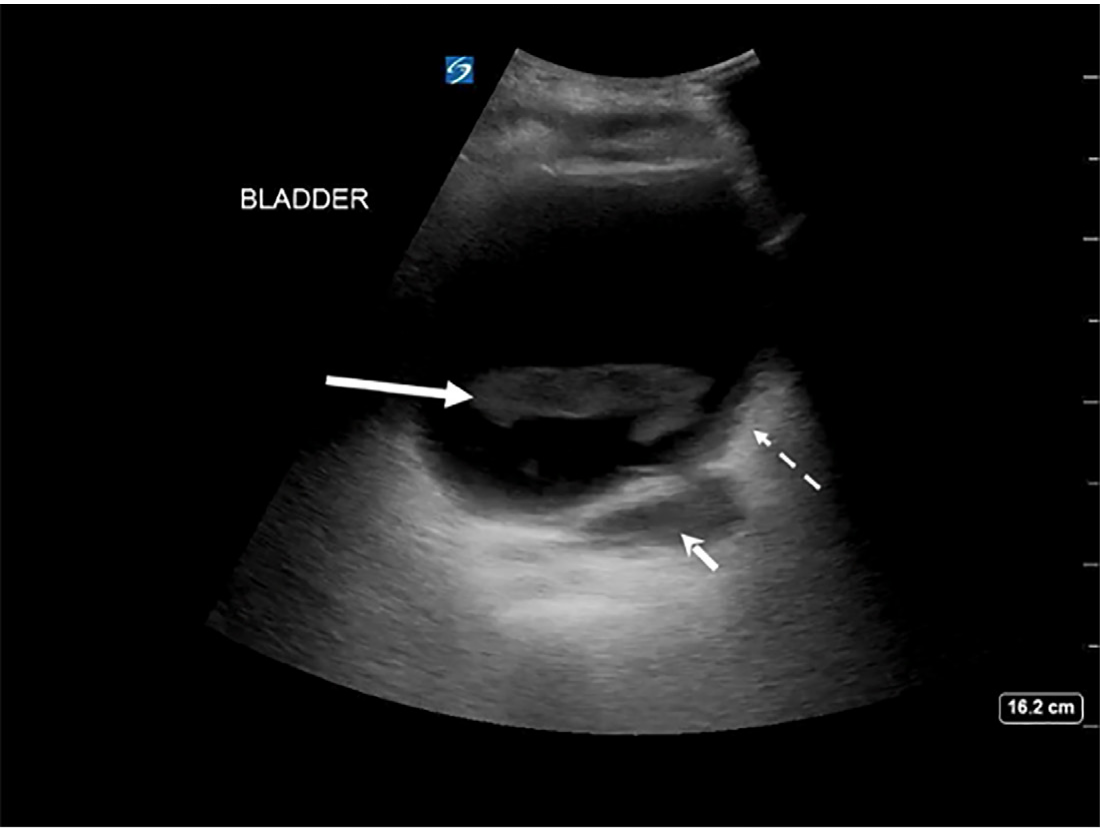
Transverse view of the bladder on point-of-care ultrasound showing echodense material in the lumen of the bladder (solid arrow), wall thickening (dashed arrow), and extravesicular fluid deep to the bladder wall (arrowhead).

To further evaluate him, a curvilinear probe was used to perform point-of-care renal and bladder ultrasound. Bladder images showed a loculated fluid collection in the superior and posterior aspect of the bladder and fluid outside the wall of the bladder, as well as hyperechoic dependent densities within the bladder lumen (Images 1 and 2).

**Image 3:**
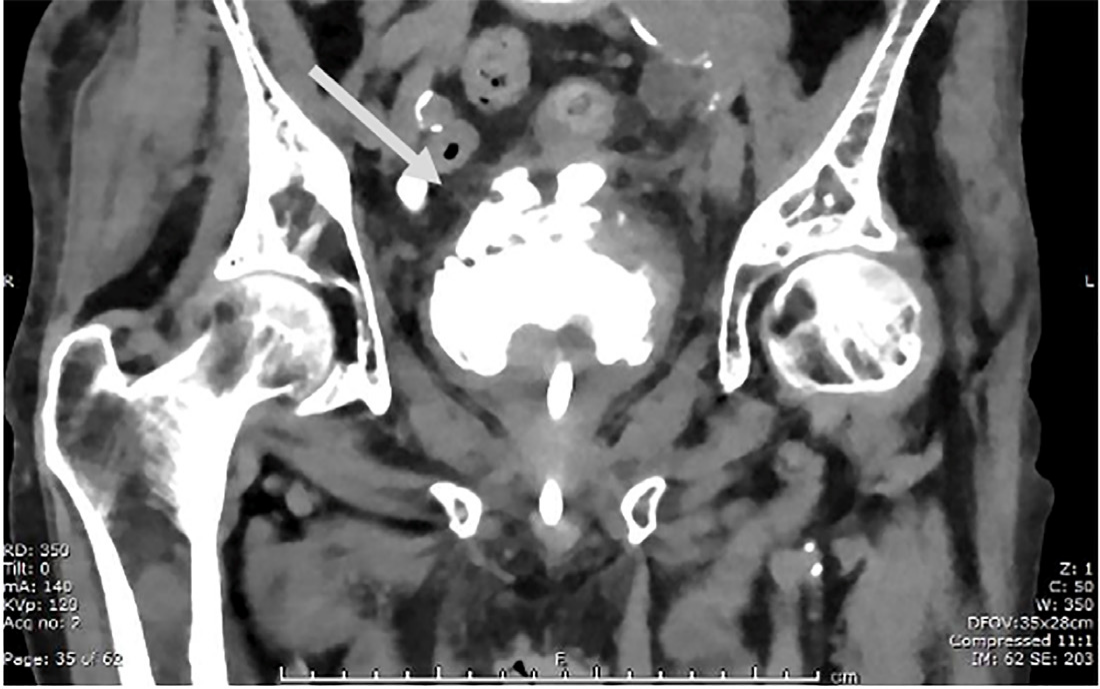
Computed tomography cystogram showing extravasated contrast contained in the extraperitoneal space (arrow).

The patient was found to be anemic with a hemoglobin of 5.5 grams per deciliter (g/dL) (reference range: 14.0–18.0 g/dL). His renal function was slightly worse than his baseline with a blood urea nitrogen of 39 milligrams/dL (11–23 mg/dL), and creatinine of 1.50 mg/dL (0.7–1.5 mg/dL). His urinalysis was positive for leukocyte esterase, nitrites, and moderate red blood cells. After placing two large-bore intravenous lines and initiating a blood transfusion, a computed tomography (CT) of the abdomen and pelvis was obtained, which showed fluid-filled loculation of the posterior aspect of the bladder measuring 9.5 centimeters, suspicious for a partially contained bladder rupture. Urology was consulted and obtained CT cystogram, which demonstrated a partially contained bladder rupture, without extension of bladder contents into the peritoneum (Image 3). Urology recommended keeping a Foley catheter in place for three weeks with repeat imaging at that time.

## DISCUSSION

Bladder rupture is commonly associated with blunt abdominal trauma or surgical complication.^2^ It is a rare complication of Foley catheter insertion.^3^ Bladder rupture is generally categorized as either intraperitoneal or extraperitoneal, or combined.^2^,^4^ It usually presents as acute abdominal pain, typically in the setting of trauma or after surgery. Retrograde cystography, whether plain film or CT, is the gold standard imaging modality to diagnose bladder rupture. However, point-of-care ultrasound (POCUS) offers a rapid alternative to assess bladder injury. The non-traumatized bladder is easily recognized on ultrasound as a well-circumscribed area of anechoic fluid in roughly a rectangular shape in the transverse plane, sometimes described as having the shape of a piece of toast.

**CPC-EM Capsule**

What do we already know about this clinical entity?

*Bladder injuries are commonly associated with blunt abdominal injury. There are only few cases that reported bladder injuries with self-catheterization*.

What is the major impact of the image(s)?

*These images show that performing a point-of-care (POC) ultrasound can easily detect bladder injuries in the right clinical settings*.

How might this improve emergency medicine practice?

*POC ultrasound can expedite the diagnosis of bladder rupture*.

Bladder rupture is recognized as an irregular-shaped bladder with free fluid adjacent to the bladder, or fluid in the right or left upper abdominal quadrants. The patient in the case had been self-catheterizing up to five times per day, increasing his risk of iatrogenic bladder injury. Clinicians should have a high yield of suspicion of bladder rupture in cases of self-catheterization. When used in the right context POCUS is a useful bedside tool to help clinicians diagnose this condition.
